# *Klebsiella pneumoniae* in Singapore: Hypervirulent Infections and the Carbapenemase Threat

**DOI:** 10.3389/fcimb.2017.00515

**Published:** 2017-12-12

**Authors:** Ka Lip Chew, Raymond T. P. Lin, Jeanette W. P. Teo

**Affiliations:** ^1^Department of Laboratory Medicine, National University Hospital, Singapore, Singapore; ^2^National Public Health Laboratory, Ministry of Health of Singapore, Singapore, Singapore

**Keywords:** *Enterobacteriaceae*, hypervirulent *Klebsiella pneumoniae* (hvKP), metastasis, antibiotic prophylaxis, resistance mechanisms

## Abstract

*Klebsiella pneumoniae* remains a major pathogen responsible for localized infections such as cystitis and pneumonia, and disseminated infections that may result in severe sepsis and death. Invasive disease such as liver abscesses and endogenous endophthalmitis are associated with capsular serotypes K1 and K2. These infections require a prolonged course of antimicrobial treatment which has evolved over the years from inpatient treatment to outpatient parenteral antibiotic therapy. The emergence of plasmid-mediated resistance began with extended-spectrum β-lactamases (ESBLs) and AmpC β-lactamases. This was followed by carbapenemase genes and now plasmid transmissible colistin resistance (*mcr*), thus limiting viable treatment options. Plasmid-mediated carbapenemase production in Singapore was first reported in 1996. Carbapenemase production has since become the predominant mechanism of carbapenem resistance and incidence rates continue to increase over time. Although carbapenemases can occur in all *Enterobacteriaceae, K. pneumoniae* are the most common carrier of carbapenemase genes. Alternative treatment options are urgently required before the simplest infections, let alone invasive infections are left potentially untreatable. Clinical management requires guidance from robust laboratory testing methods to optimize patient outcomes. We explore past and present trends in treatment of *K. pneumoniae* infections, and discuss future treatment options and gaps in knowledge for further study.

## Introduction

*Klebsiella pneumoniae* (*Kp*) is an *Enterobacteriaceae* which forms part of the gastrointestinal flora in humans. Despite being part of normal flora, they carry a variety of virulence genes and have the capacity to acquire a versatile armament of antibiotic resistance genes. As a result, *Kp* can cause both localized and disseminated infections in various settings. The clinical manifestations of *Kp* infections in Singapore are no different from neighboring countries. It has been recognized as a cause of community-acquired and hospital-acquired infections manifesting as bacteraemia, urinary tract, respiratory tract, abscesses, and hepatobiliary infections (Lee et al., 1994, 1996; Tan et al., 1998; Stebbings et al., 1999; Tay et al., 2010; Bahadin et al., 2011). The versatility of this species has given it great success in producing a multitude of clinical infections resulting in morbidity and mortality. Although the overall incidence of *Kp* infections is not monitored regularly, they are among the most commonly identified pathogen in various clinical syndromes across multiple institutions (Table 1). Reported mortality rates of *Kp* bacteraemia in Singapore ranged from 20 to 26% (Lee et al., 1994; Sng et al., 2008; Ng et al., 2015; Tan et al., 2017). We review the history of this pathogen in Singapore with a focus on hypervirulent strains, and discuss the growing problem of drug resistance and the implications for medical management now and in the future.

**Table 1:** Clinical reports of *Klebsiella pneumoniae* infections in Singapore.

**Year of Publication**	**Patient description**	***Kp* cases**	***Kp*-associated mortality (%)**	**Other Notes**	**Ref**.
**BACTERAEMIA**					
1994	Patients with *Kp* bacteraemia	101	26	Source of bacteraemia unknown (33%), Liver abscess (17%), UTI (29%) Pneumonia (9%), HBS (3%), Abscess other than liver (10%)	Lee et al., 1994
2008	Patients with *Kp* bacteraemia	133	20.3	HBS 38.3% including liver abscess in 18.0%, Pneumonia (13.3%), UTI (28.1%), Unknown source (14.8%), Others (5.5%) 5/133 (3.8%) developed endopthalmitis, all of whom had liver abscesses	Sng et al., 2008
2015	Patients with *Kp* bacteraemia	134	23.1	HBS 36.3%, including liver abscess in 14.2%, Respiratory 20.1%, No differences in mortality for cefazolin compared to 3rd generation cephalosporin	Ng et al., 2015
2017	Patients with *Kp bacteraemia*	129	25.6	HBS 34.1% including liver abscess in 16.3%, Pneumonia 18.6%, Pyelonephritis 11.6%, Cystitis 16.3%, Other intraabdominal source 8.5%, Meningitis 0.8%, Others 3.9%, Unknown 6.2%	Tan et al., 2017
**PNEUMONIA**					
1996	Patients with CAP admitted to ICU	9/61 (15%)	N/A	*Kp* was the most commonly identified pathogen	Lee et al., 1996
1998	Patients with CAP admitted to ICU	5/57 (9%)	3/5 (60%)	*Kp* was the third most commonly isolated pathogen (Tied with *Staphylococcus aureus*)	Tan et al., 1998
1999	Patients with HAP admitted to ICU	6/24 (25%)^*^	N/A	*Kp* was the most commonly identified pathogen	Stebbings et al., 1999
**URINARY TRACT INFECTIONS**					
2010	ICU patients with UTI acquired during ICU admission	3/35 (8.6%)	N/A	*Kp* was the second most commonly isolated pathogen (Tied with *Escherichia coli*)	Tay et al., 2010
2011	Community clinic patients with UTI	29/333 (8.7%)	N/A	*Kp* was the second most commonly isolated pathogen	Bahadin et al., 2011
**LIVER ABSCESS**					
2005	Review of patients with liver abscess, comparing PD vs SD	51/80 (63.8%) PD: 24/36 (66.7%) SD: 27/44 (61.4%)	Overall: 3/80 (3.8%), PD: 1/36 (2.8%), SD: 2/44 (4.5%) *Kp* specific rates N/A	*Kp* was the most commonly identified pathogen Similar treatment outcomes, except for fewer treatment failures in SD (3/44) compared to PD (10/36)	Tan et al., 2005
2013	Review of patients with *Kp* liver abscess undergoing treatment in OPAT	109/205 (53%)	0%	*Kp* was the most commonly identified pathogen Delayed response in 29/109 (27%) patients, associated with abscess >5 cm Three require readmission	Chan et al., 2013
2015	Adults with pyogenic liver abscess	292/741 (39%) of positive fluid cultures 220/741 (30%) of positive blood cultures	N/A	*Kp* was the most commonly identified pathogen	Zhi et al., 2015
**MENINGITIS**					
2002	Reviewed cases of meningitis/ meningoencephalitis	2/269 (0.7%) of clinical cases 2/15 (13.3%) culture positive cases	N/A	Only 15 cases were culture positive. The breakdown of positive cultures are as follows: 4 *Streptococcus pneumoniae*, 3 each of *Streptococcus agalactiae* and *Neisseria meningitidis*, 2 each of *Kp* and *Listeria monocytogenes*, and 1 *Streptococcus suis*	Chan et al., 2002
**ENDOGENOUS ENDOPHTHALMITIS**					
2000	Reviewed cases of endogenous bacterial endopthalmitis	16/27 (59.3%)	N/A	11 of 16 (68.8%) *Kp* cases were associated with liver abscess	Wong et al., 2000
2011	Consecutive *Kp* endogenous endopthalmitis cases reviewed	61 patients (71 eyes)	N/A	47/61 (77.5%) were associated with liver abscess 54/71 (80.2%) eyes had poor vision (VA worse than 4/200) 19/71 (26.8%) required evisceration	Ang et al., 2011

*CAP, Community-acquired pneumonia; ICU, Intensive care unit; HAP, Hospital-acquired pneumonia; UTI, Urinary tract infection; HBS, Hepatobiliary sepsis; OPAT, Outpatient parenteral antibiotic therapy; PD, Percutaneous drainage; SD, Surgical drainage; VA, Visual acuity*.

* *One patient with Kp had multiple organisms cultured*.

## Hypervirulent *Klebsiella pneumoniae* in singapore

### Characteristics of hypervirulent *Kp*

*Kp* is increasingly recognized as a cause of liver abscesses in the Asia Pacific region (Siu et al., 2012). The isolates associated with these infections are often referred to as *hypervirulent Klebsiella pneumoniae* (*hvKp*) due to the propensity for causing serious infections in healthy immunocompetent patients and dissemination throughout various sites. Several classical traits have been associated with *hvKp* as compared to classical *Kp* such as a hypermucoviscous phenotype, positive “string” test, predominance of K1 and K2 capsule type, and carriage of multiple virulence genes (Siu et al., 2012; Lee et al., 2016; Tan et al., 2017).

Although initial reports of *hvKp* were from Taiwan and South Korea, Singapore is also similarly affected (Lin et al., 2012; Zhi et al., 2015). Local *hvKp* strains have features analogous to isolates found in other parts of the world including K1 capsule type predominance followed by K2 (Lee et al., 2016; Tan et al., 2017). These K1 capsule types were typically associated with sequence type (ST) 23. The molecular epidemiology of *hvKP* STs appears to be consistent throughout the years, with ST23 found in early studies (Siu et al., 2011; Lee et al., 2016). K1/ST23 were also more likely to harbor virulence genes related to expression of the hypermucoviscous phenotype and iron uptake. *In vivo* studies with mouse models also suggest that strains of K1 capsule type were more virulent compared to non-K1 strains (Lee et al., 2016). K2 and non-K1-K2 capsule types had a greater genetic diversity in terms of ST, and were less likely to possess virulence genes (*kfu, irp, iuc, iro, rmpA)* compared to K1 capsule types (Lee et al., 2016). These virulence genes collectively contribute to pathogenesis as there is no single outstanding phenotypic or genotypic feature to which pathogenesis can be fully attributed to. Although hypermucoviscocity is associated with virulence, non-hypermucoviscous isolates may still cause disseminated disease (Lee et al., 2016; Catalán-nájera et al., 2017).

Despite the association with severe disease, not all individuals who are colonized with *hvKp* will develop disease. *Kp* is a colonizer of the human gastrointestinal tract with a higher proportion of individuals of Asian descent colonized relative to those of Western descent (Siu et al., 2012). Forty-seven out of 77 stool isolates tested from Singaporean healthy adult volunteers of Chinese ethnicity were positive for *Kp*, of which five were K1 and two were K2 capsule types (Lin et al., 2012). It is possible that infections were triggered by events that resulted in a transient bacteraemia which was then followed by dissemination (Siu et al., 2012). *HvKp* with loss of ability to translocate gastrointestinal epithelium have attenuated virulence in mouse infection models (Tu et al., 2009). However, the mechanisms have not been completely elucidated and it remains unclear why some colonized patients never develop disease.

### Invasive liver abscesses and other disseminated infections

The manifestations of *Kp* liver abscess are non-specific with fever, abdominal pain, and abnormal liver function tests being common features. Like other parts of Asia, *Kp* is the causative agent for the majority of liver abscesses in Singapore (Chan et al., 2013; Zhi et al., 2015). In the largest single-institution liver abscess case series study carried within Singapore, *Kp* was the most commonly isolated pathogen (Zhi et al., 2015). Seven hundred and forty one patients were included in this study, of whom 49% had their liver abscess aspirated or drained. Out of the 220 culture positives, 76% (167/220) were *Kp*. (Zhi et al., 2015). In a separate study, *Kp* was identified as the pathogen in 109 of 205 patients (Chan et al., 2013). Men were more commonly affected than women (Chan et al., 2013; Zhi et al., 2015). The average age of patients reported was 57 with the youngest patient reported being only 26 years of age (Chan et al., 2013). Although diabetes mellitus is a recognized risk factor for *Kp* liver abscess; this infection is not uncommon in previously well patients with no significant comorbidities. Patients with abscesses greater than 5 cm were more likely to have a delayed clinical response—either remaining symptomatic or had an elevated C-reactive-protein (CRP) at 4 weeks of treatment (Chan et al., 2013). Drainage of abscesses may be considered as an adjunct to medical management; surgical drainage of liver abscesses greater than 5 cm results in fewer treatment failures, fewer secondary procedures, and shorter length of hospital stay compared to percutaneous drainage (Tan et al., 2005).

In addition to liver abscesses, *hvKp* also have a propensity for dissemination resulting in abscesses elsewhere and other infections such as meningitis and endogenous endopthalmitis. In a review of 269 cases of meningitis or meningoencephalitis admitted between 1993 and 2000, two cases of *Kp* liver abscess were associated with meningitis (Chan et al., 2002). In a separate case series, the causative pathogen of 16 of 27 (60%) cases of endogenous endopthalmitis in Singapore was *Kp*, with hepatobiliary infection being the main source of bacteraemia (Wong et al., 2000). *Kp* was reported to be associated with poor outcomes. In another cohort study of *Kp* endophthalmitis involving 71 eyes of 61 patients, 76% suffered poor visual outcomes (Visual acuity 4/200) and 26.8% required enucleation (Ang et al., 2011). Prognostic risk factors for poor visual outcomes include presence of hypopyon, unilateral involvement, panopthalmic involvement, and the rapid onset of ocular symptoms. During an episode of bacteremia, bacteria preferentially disseminate to the vascular choroid, often presenting with a solitary choroidal abscess. If the microbial inoculum is large or the host defense is weak, *Kp* rapidly multiplies and spreads to the retina, involving the vitreous gel (Ang et al., 2011). The introduction of an ophthalmological screening program for patients with *Kp* bacteraemia was thought to have reduced the risk of progression to enucleation of eyes due to earlier diagnosis and more aggressive treatment (Ang et al., 2011). Patients were treated with intravitreal antibiotics and vitrectomy was performed if indicated. Prior to screening of at-risk patients, the ophthalmology service was involved only when patients developed ocular symptoms.

Not all patients with *Kp* bacteraemia will go on to develop endophthalmitis. Sng et al. reviewed 133 patients with *Kp* bacteraemia who received an opthalmological examination. Twenty-three of these patients had liver abscesses. Only five patients developed endopthalmitis (3.8%), all of whom had liver abscesses (5/23, 21.7%). Liver abscess and disseminated intravascular coagulation were identified as risk factors for endophthalmitis (Sng et al., 2008). Most clinicians would recommend ophthalmological screening for ocular involvement in patients with community-acquired liver abscess; the utility of screening patients with community-acquired *Kp* bacteraemia with a non-hepatobiliary or unidentified source of infection is unclear. All patients developed ophthalmic symptoms within 1 week of onset of symptoms (Sng et al., 2008). The optimum timing of an ophthalmological examination is undetermined.

### Medical management of *Klebsiella* liver abscesses

Community-acquired *hvKp* infections have typically remained susceptible to third-generation-cephalosporins and fluoroquinolones. Rarely has ESBL-producing K1 capsule type *Kp* been reported (Cheong et al., 2017). Treatment is usually of prolonged duration of 4–6 weeks in total and ceftriaxone the drug of choice for susceptible isolates. Patients were often admitted for the entirety of the treatment duration. However, management of patients with *Kp* abscesses has evolved over time. Inpatient antibiotic therapy has been followed by outpatient parenteral antibiotic therapy (OPAT) with good outcomes (Chan et al., 2013). OPAT patients are discharged home with an intravenous central-catheter (most commonly peripherally-inserted-central-catheter [PICC]), and return daily for ceftriaxone administration. Patients were able to complete their treatment without having to remain as an inpatient for the entire duration (Chan et al., 2013). Intravenous cefazolin is an appropriate alternative to ceftriaxone (Chan et al., 2013; Ng et al., 2015). Earlier studies indicated poor outcome with cefazolin use, however, this may be due to inadequate dosing of 3 g per day. The use of intravenous cefazolin at 6 g per day (administered as intravenous infusion over 24 h) demonstrated similar outcomes to intravenous ceftriaxone. Studies are now underway to compare intravenous therapy in OPAT vs. oral ciprofloxacin to further simplify treatment administration without compromising outcomes (Molton et al., 2013).

## Evolution of resistance and hospital-acquired-infections (HAI)

### Epidemiology

Although *hvKp* are problematic in community-acquired infections, non-*hvKp* also result in severe infections with impact on morbidity and mortality (Lee et al., 1994). A mortality rate of 21% was reported for community-acquired *Kp* bacteraemia. Other than the host of community-acquired-infections described earlier, *Kp* are key pathogens in hospital-acquired-infections (HAI). A recent point-prevalence study of HAI in Singapore identified *Kp* among the top three most commonly identified Gram negative cause of HAI (10.2%) behind *Pseudomonas aeruginosa* (11.5%), and *Escherichia coli* (10.4%) (Cai et al., 2017). However, *Kp* was the most frequent cause of hospital-acquired pneumonia (15.0%) and bacteraemia (20.3%). A mortality rate of 45% has been reported for hospital-acquired *Kp* bacteraemia (Lee et al., 1994). This highlights the role of this pathogen in HAI. *Kp* are also important colonizers in individuals as well the environment of both acute and subacute hospitals, and long term care facilities (Wilson et al., 2016). Infection control programs have long been aware of the need for good practices such as hand-hygiene, use of personal-protective-equipment, and isolation facilities to minimize the spread of drug-resistant organisms in healthcare settings (Wilson et al., 2016).

### Progressing from β-lactam to carbapenem resistance

Despite the generalization that hypervirulent strains tend to be antibiotic susceptible, a 1-year inter-hospital survey (years 2006 to 2007) of gram-negative bacilli suggested that 30.8 and 35.9% of *Kp* isolates were resistant to third-generation cephalosporins and fluoroquinolones respectively (Tan et al., 2008). Resistance to β-lactams is of utmost importance as they are the most commonly used group of antimicrobials. Extended-spectrum-β-lactamases (ESBLs) result in resistance to third-generation-cephalosporins and were first isolated in the 1980's in Singapore (Tse, 2008). Additionally, plasmid-mediated AmpC cephalosporinases are also prevalent at ~26%, further contributing to resistance (Tan et al., 2008). Genotypic detection of ESBLs and AmpCs by molecular approaches may not be practical due to the large number of targets. A reduction of minimum-inhibitory-concentration (MIC) to third and fourth-generation cephalosporins with the addition of β-lactamase inhibitors and lack of hydrolysis of the cephamycins is expected in ESBL positive isolates. AmpC β-lactamases are not inhibited by β-lactamase-inhibitors and typically hydrolyze third-generation-cephalosporins. AmpC enzymes do not effectively hydrolyze fourth-generation-cephalosporins such as cefepime (Jacoby, 2009). Current recommendations by European Committee on Antimicrobial Susceptibility Testing (EUCAST) and Clinical and Laboratory Standards Institute (CLSI) is that the definition of susceptibility be based on the MIC to cephalosporins. The presence or absence of resistance genes such as ESBL genes and a*mpC* does not affect the interpretation of the MIC though may still be useful for infection control purposes. However, isolation of patients with ESBL positive isolates are not practical given the proportion of ESBL colonized persons in Singapore (Young et al., 2014). Cohorting of patients colonized/infection with ESBL *Kp* has been reported in combination with other infection control measures for control of outbreak scenarios (Laurent et al., 2008; Cantey et al., 2013). The applicability of cohorting ESBL positive patients in an endemic setting is unclear. In addition, patient cohorting was introduced as a bundle with other interventions—the individual benefits of cohorting is not determined.

The optimum treatment for ESBL positive organisms is debated particularly with regard to the appropriateness of piperacillin-tazobactam compared to carbapenems for treating serious infections. Carbapenems have been considered the treatment of choice due to almost uniform susceptibility to ESBL positive isolates (Paterson and Bonomo, 2005). Although ESBLs are inhibited by β-lactamase-inhibitors, increasing inoculum may overcome this inhibition (Paterson and Bonomo, 2005). There are concerns that infections with a high initial bacterial load would produce excess β-lactamase and overcome the β-lactamase-inhibitors, resulting in treatment failure despite *in-vitro* susceptibility. Retrospective data from Singapore suggest that empiric treatment with piperacillin-tazobactam does not result in worse outcomes compared to carbapenems (Ng et al., 2016). *Post-hoc* analysis of six prospective cohort studies indicate that definitive treatment of patients with ESBL bacteraemia with piperacillin-tazobactam does not result in worse outcomes (Rodríguez-Ba-o et al., 2012). Hospitals in Singapore are involved in a multi-centre randomized non-inferiority trial (MERINO) to compare definitive treatment between carbapenems and piperacillin-tazobactam for third-generation-cephalosporin resistant *E. coli* and *Kp* (Harris et al., 2015).

Carbapenem-resistant-*Enterobacteriaceae* are a growing problem. Resistance may occur via multiple mechanisms including carbapenemase production. Isolates with carbapenemase production are referred to as carbapenemase-producing-*Enterobacteriaceae* (CPE) and are regarded as carbapenem-resistant. The detection of carbapenemases is important from an epidemiological perspective as they are plasmid-mediated and may be transferred horizontally between different bacterial species. The first carbapenemase detected in Singapore was IMP-1 in a *Kp* isolate in 1996 (Koh et al., 1999). CRE and CPE rates have increased since then (Koh et al., 2013; Marimuthu et al., 2017). Incidence of CRE in surveillance cultures increased from 0.76 per 100,000 in 2010–2012, to 16.0 per 100,000 patient days between 2013 and 2015 (Marimuthu et al., 2017). Although increased surveillance may contribute to this increase, the incidence of clinical cases have also increased from 2.89 per 100,000 patient-days in 2010 to 10.32 per 100,000 patient-days in 2013. Incidence was reported to have plateaued since 2013. Overall CPE incidence (clinical and surveillance cultures) have also increased over the same time period. Incidence of NDM-CPE was reported to increase from 2.9 per 100,000 patients-days in 2010 to 15.0 per 100,000 patients-days in 2013, and decreased to 7.11 per 100,000 patients-days in 2015. KPC-CPE is reported to be increasing with a highest incidence rate of 16.4 per 100,000 patient days in 2013. *Kp* are the most commonly reported CRE whether mediated by carbapenemase production or other mechanisms (Balm et al., 2013a; Marimuthu et al., 2017). Trends in the last seven years indicate that the IMPs have been displaced by internationally dominant carbapenemases like New Delhi Metallo-β-lactamase (NDM), *K. pneumoniae* carbapenemase (KPC), and OXA-48-like carbapenemases (Balm et al., 2012, 2013a,b; Koh et al., 2012, 2013; Teo et al., 2012, 2013, 2016a; Venkatachalam et al., 2012). Clonal spread of carbapenemase-producing-*Kp* have also been reported (Koh et al., 2013; Marimuthu et al., 2017). In 2012, four indistinguishable isolates carrying KPC were identified from patients spanning two separate hospitals (Koh et al., 2013). There was no epidemiological link observed suggesting undetected transmission. Although patients with previous overseas travel and hospitalization are risk factors for CRE colonization and infection, in a cohort of 203 patients with CRE, ~15% did not have these risk factors (Ling et al., 2015) making community transmission of CRE a possibility. Suspected and confirmed CPE isolated locally in clinical microbiology laboratories are referred to the National Public Health Laboratory (NPHL), Singapore, as part of a national surveillance program. Whole genome sequencing of CPE submitted to NPHL is performed as part of an ongoing effort by the CaPES (Carbapenemase-Producing *Enterobacteriaceae* in Singapore) Study Group to investigate CPE clinical and molecular epidemiology and reflects concerted efforts to understand and combat drug resistance in Singapore (Marimuthu et al., 2017).

### Non-β-lactam antimicrobials

In light of increasing β-lactam resistance among *Kp* isolates, there is increasing reliance on non-β-lactam antibiotics. Amongst carbapenem-resistant-*Klebsiella* spp. reported, more than 15% were resistant to polymyxin B or tigecycline, 25% to amikacin, and 65% to levofloxacin (Teo et al., 2016a). Variable rates (5–45.9%) of *qnr* plasmid-mediated quinolone resistance have been described in two local institutions (Deepak et al., 2009; Teo et al., 2009). Polymyxin B and colistin are often considered the last lines of therapy for multi-drug-resistant-Gram negatives, however the worldwide dissemination of plasmid-mediated colistin resistance encoded by *mcr variants (mcr-1*,−*2,-3*−*4*, and *-5*) may jeopardize their utility (Liu et al., 2016; Xavier et al., 2016; Borowiak et al., 2017; Carattoli et al., 2017; Yin et al., 2017). In Singapore, *mcr-1* has been detected in both carbapenemase-producing and carbapenem-susceptible *Enterobacteriaceae*. (Liu et al., 2016; Teo et al., 2016c,b). Though current *mcr-1* prevalence rates are a low of <3%, similar to global prevalence rates (Liassine et al., 2016; Saly et al., 2017), it remains to be seen if the rates continue to persist in this range.

## Laboratory testing and treatment in the era of carbapenem resistance

Clinical laboratory methods for the detection of carbapenem resistance and carbapenemase-producers are not without limitations. For example, CPE can demonstrate MICs within the susceptible breakpoint (Karlowsky et al., 2017) and hence be missed on phenotypic detection. These isolates are an uncommon occurrence (<1%) and at this stage the clinical implications are unclear (Tzouvelekis et al., 2012). Phenotypic carbapenem heteroresistance is a problematic issue. In a study of *Kp* KPC-producers (*n* = 8) which demonstrated susceptibility to a carbapenem (1–2 μg/ml), subpopulations with elevated MICs of >64 μg/ml were observed upon exposure to a carbapenem (Adams-Sapper et al., 2015). Such strains could contribute to treatment failure especially where bacterial density may be high and drug penetration suboptimal, resulting in induction of higher-level resistance (Adams-Sapper et al., 2015).

Optimal treatment of CPE is yet to be determined. Treatment usually comprises of combination therapy, inclusive of a carbapenem such imipenem or meropenem where the MIC remains ≤8 mg/L. Carbapenems used to treat CRE are usually combined with other non-β-lactam antibiotics based on drug susceptibility patterns. This may be any other antimicrobial for which phenotypic susceptibility is demonstrated. Alternatively, antimicrobials with intermediate susceptibility may also be considered with higher dosing and extended/continuous infusion times to optimize pharmacokinetic features (Dulhunty et al., 2013). Although combination therapy is commonly used, recent data suggests that mortality benefit occurs only in patients with severe illness (Gutiérrez-gutiérrez et al., 2017). Dual carbapenem-therapy has also been recommended such as doripenem-ertapenem combination where ertapenem is administered as a “sacrificial” carbapenem with is hydrolyzed by the carbapenemases, and allowing a separate carbapenem to exert its antimicrobial properties. *In vitro* synergy between multiple carbapenem combinations have also been reported (Poirel et al., 2016). An early case series reported treatment success in all three patients treated with ertapenem combined with either meropenem or doripenem (Giamarellou et al., 2013). However, a more recent study reported overall treatment success of only 39% for dual-carbapenem combinations (Cprek and Gallagher, 2015).

Polymyxins are drugs used for treatment of CRE. They have received renewed interest worldwide following increasing rates of multi-drug-resistant Gram negative organisms (Landman et al., 2008). Despite the polymyxins being discovered in the 40's and 50's, its nephrotoxicity and neurotoxicity was a strong deterrent to popular use (Landman et al., 2008). Colistin and polymyxin B are now increasingly used in the age of CRE. Minor structural differences between them result in significant pharmacokinetic differences (Landman et al., 2008). Both drugs have limited penetration through the blood-brain-barrier. Colistin is excreted into the urinary tract whilst polymyxin B is not. Colistin is also administered as a prodrug which is metabolized in the body to the active drug (Bergen et al., 2006). Polymyxin B is the more commonly used drug in Singapore. This has important implications because of issues related to susceptibility testing. Current recommendations are for broth microdilution (BMD) testing to be performed for colistin to determine the MIC (Eucast, 2016). The MIC results for colistin are not interchangeable with polymyxin B (Sader et al., 2015). In addition, testing with BMD is by no means practicable in routine microbiology laboratories. However, commonly used testing methods in Singapore such as Vitek 2 and Etest have demonstrated poor concordance with reference broth microdilution (BMD) for colistin (Chew et al., 2017). Very major error rates (false susceptible) were 12 and 26.1% for colistin and polymyxin B testing with Etest, and 36% for colistin testing with Vitek 2. Polymyxin testing with Vitek 2 had >90% essential agreement with BMD. However, due to limited pharmacokinetic and clinical data, there are no EUCAST or CLSI breakpoints available for polymyxin B susceptibility testing for *Enterobacteriaceae* to guide interpretation and treatment. The performance characteristics of Microscan and Sensititre also did not meet the standards recommended by CLSI (Essential agreement ≥90%, Categorical agreement ≥90%, Very major error ≤1.5%, Major error ≤3.0% (CLSI, 2017). Heteroresistance is another phenomenon which may impact on laboratory testing and clinical outcomes (Hawley et al., 2008; Landman et al., 2013). Further studies are required to determine suitable methods for routine testing and clinical correlation between laboratory susceptibility testing with clinical outcomes.

Synergy testing is another aspect of antimicrobial therapy for which there is limited guidance (Doern, 2014). Different methods have been described but are limited by lack of data on clinical correlation. Synergy testing is typically not performed with the exception of research settings. The use of hollow-fiber-infection-model (HFIM) for synergy testing has been reported for testing antibiotic combinations for synergy (Lim et al., 2015; Cai et al., 2016b). The HFIM is used to simulate the pharmacokinetics and pharmacodynamics of the tested drugs singly and in combination with time-kill studies. Thirty patients with drug-resistant Gram-negative bacterial infections were managed with combination antimicrobials based on synergistic drug testing with lower mortality rate compared to controls (Cai et al., 2016a). Further prospective studies are required to further correlate HFIM testing or other antimicrobial synergy testing methodologies with clinical outcomes. Implementation of such new systems are currently beyond the scope of routine laboratories.

Newer β-lactam-β-lactamase-inhibitor (BLBLI) combinations should also be considered for treatment of CPE. Vasoo et al. tested a collection of 177 carbapenemase-producing-*Enterobacteriaceae* of which 53 isolates were from Singapore (Vasoo et al., 2015). One hundred percent susceptibility to ceftazidime-avibactam (CAV) combination was demonstrated for class A (IMI and KPC) carbapenemase producing organisms. Ninety three percent of OXA-48-like CPE were susceptible to CAV. Activity of CAV was expectedly poor for metallo-β-lactamases, demonstrating susceptibility in only one of 11 IMP positive isolates demonstrated. All 32 NDM and 4 VIM positive CPE were resistant to CAV. Conversely, aztreonam-avibactam (AAV) combination demonstrated 100% susceptibility to all tested CPE, with the exception of two NDM CPE isolates from Singapore. A total of 26 NDM CPE from Singapore were included in this study suggesting a resistance to AAV of 7.7%. Although these are still limited numbers, these suggest that the introduction of CAV or AAV into hospital formularies will be useful to combat CPE. Activity of these agents in non-carbapenemase-producing-CRE is unclear. There is currently no published data on other BLBLI combinations such as meropenem-vaborbactam and imipenem-relebactam in CPE from Singapore.

## Author's perspective

*HvKp* possess a plethora of virulence factors and have the potential to gain various resistance genes. Although this combination of virulence and resistance is not common in Singapore, dissemination of clones with hypermucoviscous (and hypervirulent) phenotype and KPC-carbapenemase production has been reported (Andrade et al., 2014; Zhan et al., 2017). A noticeable absence of carbapenem-resistant ST23 and ST163 isolates was observed over 5 years (2010 to 2015, CaPES molecular surveillance study). Whilst reassuring, this should not invite complacency. Clinical and molecular characterization of *HvKp* may uncover new targets for efficient diagnosis and treatment (Lee et al., 2016; Catalán-nájera et al., 2017). In addition, ongoing investment in laboratory services is required to ensure that diagnoses of virulent and/or drug-resistant bacteria can be made efficiently. Susceptibility testing methods also require further refinement for routine antimicrobials as well as extension of current hospital formularies to include the latest treatment options. Focus on the end-point should not be lost—that is for better clinical outcomes for patients. *In vitro* data alone is insufficient and correlation with clinical outcome data is required. Most of the data so far available are retrospective. Prospective data is required with robust research methodologies. The numbers of patients in individual hospitals may be insufficient to provide sufficiently powered data. Future studies will require close inter-hospital collaboration within Singapore as well as with international groups.

## Author contributions

JT and KC: Contributed to the conceptualization, literature review and write-up of the manuscript; RL: contributed to the conceptualization and critically reviewed the manuscript.

### Conflict of interest statement

The authors declare that the research was conducted in the absence of any commercial or financial relationships that could be construed as a potential conflict of interest.

## References

[B1] Adams-Sapper S. Nolen S. Donzelli G. F. Lal M. Chen K. Da Silva L. H. J.. (2015). Rapid induction of high-level carbapenem resistance in heteroresistant KPC-producing *Klebsiella pneumoniae*. Antimicrob. Agents Chemother. 59, 3281–3289. 10.1128/AAC.05100-14 25801565 PMC4432212

[B2] Andrade L. N. Vitali L. Gaspar G. G. Bellissimo-Rodrigues F. Martinez R. Darini A. L. C. (2014). Expansion and evolution of a virulent, extensively drug-resistant (polymyxin B-resistant), QnrS1-, CTX-M-2-, and KPC-2-producing *Klebsiella pneumoniae* ST11 international high-risk clone. J. Clin. Microbiol. 52, 2530–2535. 10.1128/JCM.00088-14 24808234 PMC4097695

[B3] Ang M. Jap A. Chee S. (2011). Prognostic factors and outcomes in endogenous *Klebsiella pneumoniae* endophthalmitis. Am. J. Ophthalmol. 151, 338.e2–344.e2. 10.1016/j.ajo.2010.08.036 21168820

[B4] Bahadin J. Teo S. S. H. Mathew S. (2011). Aetiology of community-acquired urinary tract infection and antimicrobial susceptibility patterns of uropathogens isolated. Singapore Med. J. 52, 415–420. 21731993

[B5] Balm M. N. D. La M. V. Krishnan P. Jureen R. Lin R. T. P. Teo J. W. P. (2013a). Emergence of *Klebsiella pneumoniae* co-producing NDM-type and OXA-181 carbapenemases. Clin. Microbiol. Infect. 19, E421–E423. 10.1111/1469-0691.12247 23668475

[B6] Balm M. N. D. Ngan G. Jureen R. Lin R. T. P. Teo J. (2012). Molecular characterization of newly emerged blaKPC-2-Producing *Klebsiella pneumoniae* in Singapore. J. Clin. Microbiol. 50, 475–476. 10.1128/JCM.05914-11 22116160 PMC3264177

[B7] Balm M. N. D. Ngan G. Jureen R. Lin R. T. P. Teo J. W. P. (2013b). OXA-181-producing *Klebsiella pneumoniae* establishing in Singapore. BMC Infect. Dis. 13:58. 10.1186/1471-2334-13-58 23374756 PMC3570352

[B8] Bergen P. J. Li J. Rayner C. R. Nation R. L. (2006). Colistin methanesulfonate is an inactive prodrug of colistin against *Pseudomonas aeruginosa*. Antimicrob. Agents Chemother. 50, 1953–1958. 10.1128/AAC.00035-06 16723551 PMC1479097

[B9] Borowiak M. Fischer J. Hammerl J. A. Hendriksen R. S. Szabo I. Malorny B. (2017). Identification of a novel transposon-associated phosphoethanolamine transferase gene, mcr-5, conferring colistin resistance in d-tartrate fermenting *Salmonella enterica* subsp. enterica serovar Paratyphi, B. J. Antimicrob. Chemother. 72, 3317–3324. 10.1093/jac/dkx327 28962028

[B10] Cai B. Cai Y. Liew Y. X. Chua N. G. Teo J. Q. M. Lim T. P. (2016a). Clinical efficacy of polymyxin monotherapy vs. nonvalidated polymyxin combination therapy vs. validated polymyxin combination therapy in extensively drug-resistant gram-negative Bacillus infections. Antimicrob. Agents Chemother. 60, 4013–4022. 10.1128/AAC.03064-15 27090177 PMC4914661

[B11] Cai Y. Lim T. P. Teo J. Sasikala S. Lee W. Hong Y.. (2016b). *In vitro* activity of polymyxin B in combination with various antibiotics against extensively drug-resistant *Enterobacter cloacae* with decreased susceptibility to polymyxin B. Antimicrob. Agents Chemother. 60, 5238–5246. 10.1128/AAC.00270-16 27324776 PMC4997875

[B12] Cai Y. Venkatachalam I. Tee N. W. Tan T. Y. Kurup A. Wong S. Y.. (2017). Prevalence of healthcare-associated infections and antimicrobial use among adult inpatients in Singapore acute-care hospitals: results from the first national point prevalence survey. Clin. Infect. Dis. 64(Suppl. 2), S61–S67. 10.1093/cid/cix103 28475790

[B13] Cantey J. B. Sreeramoju P. Jaleel M. Trevi-o S. Gander R. Hynan L. S.. (2013). Prompt control of an outbreak caused by extended-spectrum β-lactamase-producing *Klebsiella Pneumoniae* in a neonatal intensive care unit. J Pediatr. 163, 672.e3–679.e3. 10.1016/j.jpeds.2013.03.001 23582136

[B14] Carattoli A. Villa L. Feudi C. Curcio L. Orsini S. Luppi A.. (2017). Novel plasmid-mediated colistin resistance mcr-4 gene in Salmonella and *Escherichia coli*, Italy 2013, Spain and Belgium, 2015 to 2016. Eurosurveillance 22:30589. 10.2807/1560-7917.ES.2017.22.31.30589 28797329 PMC5553062

[B15] Catalán-nájera J. C. Garza-ramos U. Barrios H. Catal J. C. (2017). Hypervirulence and hypermucoviscosity: two different but complementary *Klebsiella* spp. phenotypes? Virulence 8, 1111–1123. 10.1080/21505594.2017.1317412 28402698 PMC5711391

[B16] Chan D. S. G. Archuleta S. Llorin R. M. Lye D. C. Fisher D. (2013). Standardized outpatient management of *Klebsiella pneumoniae* liver abscesses. Int. J. Infect. Dis. 17, e185–e188. 10.1016/j.ijid.2012.10.002 23154175

[B17] Chan Y. C. Wilder-Smith A. Ong B. K. C. Kumarasinghe G. Wilder-Smith E. (2002). Adult community acquired bacterial meningitis in a Singaporean teaching hospital. A seven-year overview (1993-2000). Singapore Med. J. 43, 632–636. 12693768

[B18] Cheong H. S. Chung D. R. Park M. Kim S. H. Ko K. S. (2017). Emergence of an extended-spectrum β -lactamase-producing serotype K1 *Klebsiella pneumoniae* ST23 strain from Asian countries. Epidemiol. Infect. 145, 990–994. 28031071 10.1017/S0950268816003113 PMC9507823

[B19] Chew K. L. La M.-V. Lin R. T. P. Teo J. W. P. (2017). Colistin and polymyxin B susceptibility testing for carbapenem-resistant and mcr -positive Enterobacteriaceae : comparison of sensititre, microscan, vitek 2, and etest with broth microdilution. J. Clin. Microbiol. 55, 2609–2616. 10.1128/JCM.00268-17 28592552 PMC5648698

[B20] CLSI (2017). M52 Verification of Commercial Microbial Identification and Antimicrobial Susceptibility Testing Systems. Wayne, PA: Clinical and Laboratory Standards Institute.

[B21] Cprek J. B. Gallagher J. C. (2015). Ertapenem-containing double-carbapenem therapy for treatment of infections caused by carbapenem-resistant *Klebsiella pneumoniae*. Antimicrob. Agents Chemother. 60, 669–673. 26552970 10.1128/AAC.01569-15 PMC4704223

[B22] Deepak R. N. Koh T. H. Chan K. S. (2009). Plasmid-mediated quinolone resistance determinants in urinary isolates of *Escherichia coli* and *Klebsiella pneumoniae* in a Large Singapore Hospital. Ann. Acad. Med. Singapore 38, 1070–1073. 20052442

[B23] Doern C. D. (2014). When does 2 plus 2 equal 5? A review of antimicrobial synergy testing. J. Clin. Microbiol. 52, 4124–4128. 10.1128/JCM.01121-14 24920779 PMC4313275

[B24] Dulhunty J. M. Roberts J. A. Davis J. S. Webb S. A. R. Bellomo R. Gomersall C.. (2013). Continuous infusion of beta-lactam antibiotics in severe sepsis: a multicenter double-blind, randomized controlled trial. Clin. Infect. Dis. 56, 236–244. 10.1093/cid/cis856 23074313

[B25] Eucast (2016). Recommendations for MIC determination of colistin (polymyxin E) As recommended by the joint CLSI-Eucast Polymyxin Breakpoints Working Group.

[B26] Giamarellou H. Galani L. Baziaka F. Karaiskos I. (2013). Effectiveness of a double-carbapenem regimen for infections in *Klebsiella pneumoniae*. Antimicrob. Agents Chemother. 57, 2388–2390. 10.1128/AAC.02399-12 23439635 PMC3632902

[B27] Gutiérrez-gutiérrez B. Salamanca E. Cueto M. De Hsueh P. Viale P. Pa-o-pardo J. R.. (2017). Effect of appropriate combination therapy on mortality of patients with bloodstream infections due to carbapenemase-producing Enterobacteriaceae (INCREMENT): a retrospective cohort study. Lancet Infect. Dis. 17, 726–734. 10.1016/S1473-3099(17)30228-1 28442293

[B28] Harris P. Peleg A. Y. Iredell J. Ingram P. R. Miyakis S. Stewardson A. J. (2015). Meropenem vs. piperacillin-tazobactam for definitive treatment of bloodstream infections due to ceftriaxone non-susceptible *Escherichia coli* and *Klebsiella* spp (the MERINO trial): study protocol for a randomised controlled trial. Trials 16, 24 10.1186/s13063-014-0541-9 25623485 PMC4311465

[B29] Hawley J. S. Murray C. K. Jorgensen J. H. (2008). Colistin heteroresistance in Acinetobacter and its association with previous colistin therapy. Antimicrob. Agents Chemother. 52, 351–352. 10.1128/AAC.00766-07 17954699 PMC2223915

[B30] Jacoby G. A. (2009). AmpC β-Lactamases. Clin. Microbiol. Rev. 22, 161–182. 10.1128/CMR.00036-08 19136439 PMC2620637

[B31] Karlowsky J. A. Lob S. H. Kazmierczak K. M. Badal R. E. Young K. Motyl M. R. (2017). *In Vitro* Activity of Imipenem against carbapenemase-positive Enterobacteriaceae : SMART Global Surveillance Program 2008-2014. J. Clin. Microbiol. 55, 1638–1649. 10.1128/JCM.02316-16 28298454 PMC5442520

[B32] Koh T. Cao D. Chan K. Wijaya L. Low S. Lam M.. (2012). bla OXA-181 – positive Klebsiella Singapore. Emerg. Infect. Dis. 18, 1524–1525. 10.3201/eid1809.111727 22931561 PMC3437718

[B33] Koh T. H. Babini G. S. Woodford N. Sng L. H. Hall L. M. Livermore D. M. (1999). Carbapenem-hydrolysing IMP-1 beta-lactamase in *Klebsiella pneumoniae* from Singapore. Lancet 353:2162. 10382730 10.1016/s0140-6736(05)75604-x

[B34] Koh T. H. Cao D. Shan Q. Y. Bacon A. Hsu L.-Y. Ooi E. E. (2013). Acquired carbapenemases in Enterobactericeae in Singapore, 1996-2012. Pathology 45, 600–603. 10.1097/PAT.0b013e3283650b1e. 24018814

[B35] Landman D. Georgescu C. Martin D. A. Quale J. (2008). Polymyxins revisited. Clin. Microbiol. Rev. 21, 449–465. 10.1128/CMR.00006-08 18625681 PMC2493081

[B36] Landman D. Salamera J. Quale J. (2013). Irreproducible and uninterpretable polymyxin B MICs for enterobacter cloacae and enterobacter aerogenes. J. Clin. Microbiol. 51, 4106–4111. 10.1128/JCM.02129-13 24088860 PMC3838098

[B37] Laurent C. Rodriguez-Villalobos H. Rost F. Strale H. Vincent J. Deplano A.. (2008). Intensive care unit outbreak of extended-spectrum beta-lactamase-producing *Klebsiella pneumoniae* controlled by cohorting patients and reinforcing infection control measures. Infect. Control Hosp. Epidemiol. 29, 517–524. 10.1086/588004 18510461

[B38] Lee I. R. Molton J. S. Wyres K. L. Gorrie C. Wong J. Hoh C. H.. (2016). Differential host susceptibility and bacterial virulence factors driving Klebsiella liver abscess in an ethnically diverse population. Sci. Rep. 6:29316. 10.1038/srep29316 27406977 PMC4942785

[B39] Lee K. H. Hui K. P. Tan W. C. Lim T. K. (1994). Klebsiella bacteraemia: a report of 101 cases from National University Hospital, Singapore. J. Hosp. Infect. 27, 299–305. 10.1016/0195-6701(94)90117-1 7963472

[B40] Lee K. H. Hui K. P. Tan W. C. Lim T. K. (1996). Severe community-acquired pneumonia in Singapore. Singapore Med. J. 37, 374–377. 8993135

[B41] Liassine N. Assouvie L. Descombes M.-C. Tendon V. D. Kieffer N. Poirel L.. (2016). Very low prevalence of MCR-1/MCR-2 plasmid-mediated colistin resistance in urinary tract Enterobacteriaceae in Switzerland. Int. J. Infect. Dis. 51, 4–5. 10.1016/j.ijid.2016.08.008 27544715

[B42] Lim T. P. Cai Y. Hong Y. Chan E. C. Y. Suranthran S. Teo J. Q. M.. (2015). *In vitro* pharmacodynamics of various antibiotics in combination against extensively drug-resistant *Klebsiella pneumoniae*. Antimicrob. Agents Chemother. 59, 2515–2524. 10.1128/AAC.03639-14 25691628 PMC4394811

[B43] Lin Y. Siu L. K. Lin J. Chen T. Tseng C. Yeh K. (2012). Seroepidemiology of *Klebsiella pneumoniae* colonizing the intestinal tract of healthy chinese and overseas chinese adults in Asian countries. BMC Microbiol 12:13. 10.1186/1471-2180-12-13 22260182 PMC3273430

[B44] Ling M. L. Tee Y. M. Tan S. G. Amin I. M. How K. B. Tan K. Y.. (2015). Risk factors for acquisition of carbapenem resistant Enterobacteriaceae in an acute tertiary care hospital in Singapore. Antimicrob. Resist. Infect. Control 4, 26. 10.1186/s13756-015-0066-3 26106476 PMC4477303

[B45] Liu Y. Y. Wang Y. Walsh T. R. Yi L. X. Zhang R. Spencer J.. (2016). Emergence of plasmid-mediated colistin resistance mechanism MCR-1 in animals and human beings in China: a microbiological and molecular biological study. Lancet Infect. Dis. 16, 161–168. 10.1016/S1473-3099(15)00424-7 26603172

[B46] Marimuthu K. Venkatachalam I. Xin Khong W. Hsien Koh T. Pei Zhi Cherng B. Van La M. (2017). Clinical infectious diseases clinical and molecular epidemiology of carbapenem- resistant enterobacteriaceae among adult inpatients in Singapore; for the carbapenemase-producing Enterobacteriaceae in Singapore (CaPES) study group. Clin. Infect. Dis. 64(Suppl. 2), S68–S75. 10.1093/cid/cix113 28475792

[B47] Molton J. Phillips R. Gandhi M. Yoong J. Lye D. Tan T. T. (2013). Oral vs. intravenous antibiotics for patients with *Klebsiella pneumoniae* liver abscess: study protocol for a randomized controlled trial. Trials 14:364 10.1186/1745-6215-14-364 24176222 PMC4228424

[B48] Ng T. M. Khong W. X. Harris P. N. A. De P. P. Chow A. Tambyah P. A.. (2016). Empiric piperacillin-tazobactam vs. carbapenems in the treatment of bacteraemia due to extended-spectrum beta-lactamase-producing enterobacteriaceae. PLoS ONE 11:e0153696. 10.1371/journal.pone.0153696 27104951 PMC4841518

[B49] Ng T. M. Teng C. B. Lew E. L. Ling L. M. Ang B. Lye D. C. (2015). Potential for cefazolin as de-escalation therapy for *Klebsiella pneumoniae* bacteraemia. Ann. Acad. Med. Singapore 44, 571–574. 27090077

[B50] Paterson D. L. Bonomo R. A. (2005). Extended-spectrum beta-lactamases : a clinical update. Clin. Microbiol. Rev. 18, 657–686. 10.1128/CMR.18.4.657-686.2005 16223952 PMC1265908

[B51] Poirel L. Kieffer N. Nordmann P. (2016). *In vitro* evaluation of dual carbapenem combinations against carbapenemase-producing Enterobacteriaceae. J. Antimicrob. Chemother. 71, 156–161. 10.1093/jac/dkv294 26416781

[B52] Rodríguez-Ba-o J. Navarro M. D. Retamar P. Picón E. Pascual Á. (2012). β-Lactam/β-lactam inhibitor combinations for the treatment of bacteremia due to extended-spectrum β-lactamase-producing *Escherichia coli*: a *post hoc* analysis of prospective cohorts. Clin. Infect. Dis. 54, 167–174. 10.1093/cid/cir790 22057701

[B53] Sader H. S. Rhomberg P. R. Farrell D. J. Jones R. N. (2015). Differences in potency and categorical agreement between colistin and polymyxin B when testing 15,377 clinical strains collected worldwide. Diagn. Microbiol. Infect. Dis. 83, 379–381. 10.1016/j.diagmicrobio.2015.08.013 26415906

[B54] Saly M. Jayol A. Poirel L. Megraud F. Nordmann P. Dubois V. (2017). Prevalence of faecal carriage of colistin-resistant Gram-negative rods in a university hospital in western France, (2016). J. Med. Microbiol. 66, 842–843. 10.1099/jmm.0.000497 28598315

[B55] Siu L. K. Fung C. P. Chang F. Y. Lee N. Yeh K. M. Koh T. H.. (2011). Molecular typing and virulence analysis of serotype K1 *Klebsiella pneumoniae* strains isolated from liver abscess patients and stool samples from noninfectious subjects in Hong Kong, Singapore, and Taiwan. J. Clin. Microbiol. 49, 3761–3765. 10.1128/JCM.00977-11 21900521 PMC3209116

[B56] Siu L. K. Yeh K. Lin J. Fung C. Chang F. (2012). *Klebsiella pneumoniae* liver abscess : a new invasive syndrome. Lancet Infect. Dis. 12, 881–887. 10.1016/S1473-3099(12)70205-0 23099082

[B57] Sng C. C. A. Jap A. Chan Y. H. Chee S. (2008). Risk factors for endogenous Klebsiella endophthalmitis in patients with *Klebsiella bacteraemia*: a case-control study. Br. J. Ophthalmol. 92, 673–677. 10.1136/bjo.2007.132522 18245273

[B58] Stebbings A. E. L. Ti T. Y. Tan W. C. (1999). Hospital acquired pneumonia in the medical intensive care unit - A prospective study. Singapore Med. J. 40, 508–512. 10572489

[B59] Tan T. Y. Ng S. Y. Teo L. Koh Y. Teok C. H. (2008). Detection of plasmid-mediated AmpC in *Escherichia coli, Klebsiella pneumoniae* and *Proteus mirabilis*. J. Clin. Pathol. 61, 642–644. 10.1136/jcp.2007.053470 18057079

[B60] Tan T. Y. Ong M. Cheng Y. Ng L. S. Y. (2017). Hypermucoviscosity, rmpA, and aerobactin are associated with community-acquired Klebsiella pneumoniae bacteremic isolates causing liver abscess in Singapore. J. Microbiol. Immunol. Infect. [Epub ahead of print]. 10.1016/j.jmii.2017.07.003 28736222

[B61] Tan Y. Chung A. Y. Chow P. K. Cheow P. Wong W.-K. Ooi L. L.. (2005). An appraisal of surgical and percutaneous drainage for pyogenic liver abscesses larger than 5 cm. Ann. Surg. 241, 485–490. 10.1097/01.sla.0000154265.14006.47 15729072 PMC1356988

[B62] Tan Y. K. Khoo K. L. Chin S. P. Ong Y. Y. (1998). Aetiology and outcome of severe community-acquired pneumonia in Singapore. Eur. Respir. J. 12, 113–115. 10.1183/09031936.98.12010113 9701424

[B63] Tay M. K. X. Lee J. Y. C. Wee I. Y. J. Oh H. M. L. (2010). Evaluation of intensive care unit-acquired urinary tract infections in Singapore. Ann. Acad. Med. Singapore 39, 460–465. 20625622

[B64] Teo J. Cai Y. Lim T.-P. Tan T. Kwa A. (2016a). Carbapenem resistance in gram-negative bacteria: the not-so-little problem in the little red dot. Microorganisms 4:13. 10.3390/microorganisms4010013 27681907 PMC5029518

[B65] Teo J. Ngan G. Balm M. Jureen R. Krishnan P. Lin R. (2012). Molecular characterization of NDM-1 producing Enterobacteriaceae isolates in Singapore hospitals. West Pac. Surveill. Response 3, 19–24. 10.5365/wpsar.2011.2.4.010 23908903 PMC3729072

[B66] Teo J. Q. Ong R. T. Xia E. Koh T. Khor C. Lee S. J.. (2016c). mcr-1 in Multidrug-Resistant blaKPC-2 -producing clinical enterobacteriaceae isolates in Singapore. Antimicrob. Agents Chemother. 60, 6435–6437. 10.1128/AAC.00804-16 27503652 PMC5038244

[B67] Teo J. W. Chew K. L. Lin R. T. (2016b). Transmissible colistin resistance encoded by mcr-1 detected in clinical Enterobacteriaceae isolates in Singapore. Emerg. Microbes. Infect. 5:e87. 10.1038/emi.2016.85 27530747 PMC5034101

[B68] Teo J. W. P. Kurup A. Lin R. T. P. Hsien K. T. (2013). Emergence of clinical *Klebsiella pneumoniae* producing OXA-232 carbapenemase in Singapore. New Microbes New Infect. 1, 13–15. 10.1002/2052-2975.4 25356318 PMC4184486

[B69] Teo J. W. P. Ng K. Y. Lin R. T. P. (2009). Detection and genetic characterisation of qnrB in hospital isolates of *Klebsiella pneumoniae* in Singapore. Int. J. Antimicrob. Agents 33, 177–180. 10.1016/j.ijantimicag.2008.08.019 18993034

[B70] Tse H. K. (2008). Gram-negative resistance in Singapore: a historical perspective. Ann. Acad. Med. Singapore. 37, 847–854. 19037519

[B71] Tu Y. C. Lu M. C. Chiang M. K. Huang S. P. Peng H. L. Chang H. Y.. (2009). Genetic requirements for Klebsiella pneumoniae-induced liver abscess in an oral infection model. Infect. Immun. 77, 2657–2671. 10.1128/IAI.01523-08 19433545 PMC2708586

[B72] Tzouvelekis L. S. Markogiannakis A. Psichogiou M. Tassios P. T. Daikos G. L. (2012). Carbapenemases in *Klebsiella pneumoniae* and other Enterobacteriaceae: an evolving crisis of global dimensions. Clin. Microbiol. Rev. 25, 682–707. 10.1128/CMR.05035-11 23034326 PMC3485753

[B73] Vasoo S. Cunningham S. A. Cole N. C. Kohner P. C. Menon S. R. Krause K. M.. (2015). *In Vitro* activities of ceftazidime-avibactam, aztreonam-avibactam, and a panel of older and contemporary antimicrobial agents against carbapenemase-producing gram-negative bacilli. Antimicrob. Agents Chemother. 59, 7842–7846. 10.1128/AAC.02019-15 26392487 PMC4649150

[B74] Venkatachalam I. Teo J. Balm M. N. D. Fisher D. A. Jureen R. Lin R. T. P. (2012). Klebsiella pneumonia carbapenemase-producing enterobacteria in hospital, Singapore. Emerging Infect. Dis. 18, 1381–1383. 10.3201/eid1808.110893 22840461 PMC3414009

[B75] Wilson A. P. R. Livermore D. M. Otter J. A. Warren R. E. Jenks P. Enoch D. A.. (2016). Prevention and control of multi-drug-resistant Gram-negative bacteria: Recommendations from a Joint Working Party. J. Hosp. Infect. 92, S1–S44. 10.1016/j.jhin.2015.08.007 26598314

[B76] Wong J.-S. Chan T.-K. Lee H.-M. Chee S.-P. (2000). Endogenous bacterial endophthalmitis: an east Asian experience and reappraisal of a severe ocular affliction. Ophthalmology 107, 1483–1491. 10.1016/S0161-6420(00)00216-5 10919895

[B77] Xavier B. B. Lammens C. Ruhal R. Malhotra-Kumar S. Butaye P. Goossens H.. (2016). Identification of a novel plasmid-mediated colistinresistance gene, mcr-2, in Escherichia coli, Belgium, June. *Eurosurveillance* 21. 10.2807/1560-7917.ES.2016.21.27.30280 27416987

[B78] Yin W. Li H. Shen Y. Liu Z. Wang S. Shen Z. (2017). Novel Plasmid-mediated colistin resistance gene mcr-3 in *Escherichia coli*. MBio 8, e00543–e00517. 10.1128/mBio.00543-17 28655818 PMC5487729

[B79] Young B. E. Lye D. C. Krishnan P. Chan S. Leo Y. (2014). A prospective observational study of the prevalence and risk factors for colonization by antibiotic resistant bacteria in patients at admission to hospital in Singapore. BMC Infect. Dis. 14:298. 10.1186/1471-2334-14-298 24889720 PMC4057577

[B80] Zhan L. Wang S. Guo Y. Jin Y. Duan J. Hao Z.. (2017). Outbreak by hypermucoviscous *Klebsiella pneumoniae* ST11 isolates with carbapenem resistance in a tertiary hospital in China. Front. Cell. Infect. Microbiol. 7:182. 10.3389/fcimb.2017.00182 28560183 PMC5432538

[B81] Zhi J. Lo W. Jia J. Leow J. Ling P. Ng F. (2015). Predictors of therapy failure in a series of 741 adult pyogenic liver abscesses. J. Hepatobililary Pancreat. Sci. 22, 156–165. 10.1002/jhbp.174 25339111

